# Clinical Features, Treatment and Outcome of Mucosa-Associated Lymphoid Tissue (MALT) Lymphoma of the Ocular Adnexa: Single Center Experience of 60 Patients

**DOI:** 10.1371/journal.pone.0104004

**Published:** 2014-07-31

**Authors:** Barbara Kiesewetter, Julius Lukas, Andreas Kuchar, Marius E. Mayerhoefer, Berthold Streubel, Heimo Lagler, Leonhard Müllauer, Stefan Wöhrer, Julia Fischbach, Markus Raderer

**Affiliations:** 1 Department of Medicine I, Division of Oncology, Medical University of Vienna, Vienna, Austria; 2 Department of Ophthalmology, Medical University of Vienna, Vienna, Austria; 3 Department of Radiology, Medical University of Vienna, Vienna, Austria; 4 Department of Pathology, Medical University of Vienna, Vienna, Austria; 5 Department of Medicine I, Division of Infectious Diseases and Tropical Medicine, Medical University of Vienna, Vienna, Austria; 6 Department of Medicine I, Division of Bone Marrow Transplantation, Medical University of Vienna, Vienna, Austria; University of Nebraska Medical Center, United States of America

## Abstract

**Background:**

Orbital marginal zone B-cell lymphoma (OAML) constitutes for the most frequent diagnosis in orbital lymphoma. Relatively little data, however, have been reported in larger cohorts of patients staged in a uniform way and no therapy standard exists to date.

**Material and Methods:**

We have retrospectively analyzed 60 patients diagnosed and treated at our institution 1999–2012. Median age at diagnosis was 64 years (IQR 51–75) and follow-up time 43 months (IQR 16–92). All patients had undergone uniform extensive staging and histological diagnosis was made by a reference pathologist according to the WHO classification.

**Results:**

The majority of patients presented with stage IE (n = 40/60, 67%), three had IIE/IIIE and the remaining 17 stage IVE. Seven patients with IVE had bilateral orbital disease whereas the others showed involvement of further organs. Treatment data were available in 58 patients. Local treatment with radiotherapy (14/58, 24%) or surgery (3/58, 5%) resulted in response in 82% of patients. A total of 26 patients (45%) received systemic treatment with a response rate of 85%. Nine patients received antibiotics as initial therapy; response rate was 38%. Watchful-waiting was the initial approach in 6/58 patients. In total 28/58 patients (48%) progressed and were given further therapy. Median time-to-progression in this cohort was 20 months (IQR 9–39). There was no difference in time-to-progression after first-line therapy between the different therapy arms (p = 0.14). Elevated beta-2-microglobulin, plasmacytic differentiation, autoimmune disorder and site of lymphoma were not associated with a higher risk for progress.

**Conclusion:**

Our data underscore the excellent prognosis of OAML irrespective of initial therapy, as there was no significant difference in time-to-progression and response between local or systemic therapy. In the absence of randomized trials, the least toxic individual approach should be chosen for OAML.

## Introduction

Lymphomas of the ocular adnexa including orbit, extraocular muscles, conjunctiva, eyelids and lacrimal gland constitute a histologically heterogeneous group with a rising incidence in the last decades. Common subtypes are diffuse large B-cell lymphoma (DLBCL) (about 9%) and follicular lymphoma (10–20%), while the most frequently diagnosed subtype is lymphoma of the mucosa associated lymphoid tissue (MALT lymphoma) [1]–[3].

Thought to originate from mature B-cells in the marginal zone of MALT, this distinct indolent lymphoma is most prominent for gastric (50%) involvement, but also arises in other extranodal locations including lung, head and neck with salivary glands, skin, and, as highlighted, the ocular adnexa, referred to as ocular adnexal marginal zone B-cell lymphoma (OAML) [4]. Various triggers including Helicobacter pylori (HP) in gastric MALT lymphoma [5] or autoimmune disorders e.g. Sjögren’s syndrome in parotid gland MALT lymphoma and chronic autoimmune thyroiditis (Hashimoto’s disease) for thyroid, but also gastric MALT lymphomas have been identified [6], [7]. In analogy to Helicobacter pylori, Chlamydophila Psittaci (CP) has also been defined as a potential pathogen in the development of OAML, despite a strong geographic variation of incidence [8], [9]. While antibiotic eradication of HP can lead to long term complete remission in approximately 80% of gastric MALT lymphoma and has universally been adopted as first line therapy, various therapeutic approaches including radiotherapy and different immuno-/chemotherapy protocols have been used for second line therapy in HP-refractory gastric MALT lymphoma or for treatment of non-gastric MALT lymphoma [10], in the latter mostly in an uncontrolled way.

In case of OAML, excellent local control using radiotherapy has repeatedly been reported, but ophthalmologic outcomes may be unfavourable as decreased visual acuity and deterioration of lens opacity will occur in a dose dependent manner following radiation [11]–[13]. As MALT lymphoma can present in a disseminated way in up to one third of patients [14], depending on the depth of staging applied, systemic therapy using various chemotherapies with or without the monoclonal anti-CD20 antibody rituximab seems to be a reasonable option and various combinations have successfully been applied [11], [12], [15]–[17]. Furthermore, also immunotherapy or immunomodulatory therapy [18], [19], immunoradiotherapy with yttrium-90-ibritumomab tiuxetan [20], [21] or a primary wait-and-see strategy [22] have been reported as reasonable therapeutic approaches. Antibiotic treatment with either doxycycline or clarithromycin targeting CP has recently been tested in several prospective and retrospective series, and has achieved overall response rates (ORR) up to 65% [23]–[27]. While reported in only non-randomized trials, this suggests a potential application of antibiotics as first line therapy in OAML as well.

Relatively little data, however, have been reported in larger cohorts of patients staged in a uniform way, and no randomized trials exist to put the currently data on local and systemic therapy into perspective. In view of this, we have retrospectively analysed all patients with OAML diagnosed, staged and treated at our institution to assess clinical characteristics and treatment outcome of this relatively large cohort of Austrian patients with OAML.

## Materials and Methods

A retrospective analysis of all MALT lymphoma patients diagnosed and treated at our tertiary referral center between January 1999 and December 2012 was performed to identify patients with OAML. No additional informed consent was obtained as all patients treated at our clinic sign informed consent at the very first visit that their anonymized routine data may be used for scientific purpose by clinic staff. Thus, the analysis had been approved by the Ethical Board of the Medical University of Vienna without explicitely requiring a specific informed consent. Routine medical records were used as source documents which are available to all doctors at the department. All patients were numbered sequentially, anonymized and de-identified prior to analysis by doctors who had also been involved in the routine care of these patients. Extracted data were stored and analyzed on a PC with access restrictions at the Department of Internal Medicine I/Clinical Division of Oncology.

In all patients with OAML, tissue samples of orbital lesions were histologically examined according to the World Health Organization (WHO) classification for tumors of hematologic and lymphoid tissues [4] including adequate immunophenotyping on paraffin-embedded specimens, i.e. CD20+CD5-CD10-cyclinD1- and demonstration of light chain restriction, as also highlighted in the WHO-classification. Furthermore presence or absence of plasmacytic differentiation was assessed according to the criteria defined by the WHO-classification. All diagnoses were (re-)assessed and confirmed by a reference haematopathologist (L.M.).

Primary information abstracted from patient’s records was initial localization of MALT lymphoma and extent of disease as defined by the Ann Arbor staging system. All patients underwent an extensive staging-procedure including magnetic resonance imaging (MRI) of neurocranium and orbit, standardized orbital echography, imaging of salivary glands either using sonography, CT or MRI, sonography of axillary, cervical and inguinal lymph nodes and CT scan of thorax/abdomen [28]. In the initial 20 patients, a GI-work up consisting of gastroscopy, upper GI-endosonography and a colonoscopy was performed, as well as a bone marrow biopsy. Initial laboratory work-up included a complete blood count, lactate dehydrogenase levels, serum electrophoreses, immunofixation for detection of paraproteins, screening for autoimmune diseases and for hepatitis A, B and C virus and serological testing for HP. Basic information of patients including sex, age, performance status (PS) at initial diagnosis, international prognostic index (IPI) score and follow-up time were recorded. First line treatment and consecutive lines of therapy if applicable were assessed, and radiological outcome according to RECIST 1.1 [29] was re-assessed by a reference radiologist as defined for complete remission (CR), partial remission (PR), stable disease (SD) or progressive disease (PD) in patients with measurable disease. In patients with lymphoma restricted to the conjunctiva and thus potential absence of measurable lesions by means of CT or MRI, findings of standardized orbital A-scan echography and clinical ophthalmological assessment as documented in patient records were used for response evaluation. Corresponding time to progression (TTP) was computed in patients relapsing or progressing. In order to simplify reading and statistical evaluation of data, patients were stratified into treatment subgroups for statistical analysis, i.e. antibiotic therapy, radiotherapy, immuno-/chemotherapy, surgery and wait-and-see. Also patients included in clinical trials with agents not approved for therapy of MALT lymphoma with a diagnosis of MALT lymphoma of the ocular adnexa were analysed and evaluated in this report.

Statistical analysis: The association of binary variables was calculated using the chi square-test. Means between two groups were compared with the student’s t-test. Means between more than two groups were compared with the ANOVA-test and post hoc-analyzes were done with the Duncan-test. P-values below 0.05 were considered statistically significant. Estimated time to progression and estimated time to next treatment was calculated and plotted with the Kaplan Meier-method. Differences among the groups were tested for significance with the log-rank test.

## Results

### Patient characteristics

A total of 60 patients with histologically verified OAML diagnosed and treated at our institution between 1999 and 2012 were identified and included in the analysis. The median age at diagnosis was 64 years with an interquartile range (IQR) of 51–75 years. Gender was almost equally distributed with 33/60 (55%) being female and 27/60 (45%) patients being male, and the median follow-up time from initial diagnosis on was 43 months (IQR 16–92).

In all patients, ocular adnexal manifestations/symptoms had led to the clinical suspicion of lymphoma with subsequent biopsy performed by an ophthalmologist. The most frequent site of OAML was the conjunctiva (22/60, 37%), followed by lymphoma of the lacrimal gland (18/60, 30%) and intraorbital manifestations, i.e. diffuse involvement of the extraconal part of the orbit or lymphoma of the orbital muscles (20/60, 33%). The majority of patients presented with localized disease i.e. stage IE according to Ann Arbor (40/60, 67%), one further patient with histologically verified cervical lymph node involvement with stage IIE (2%) while two patients had stage IIIE (3%) and the remaining 17 (28%) stage IVE disease. Seven of 17 patients with stage IVE presented with bilateral disease whereas the other 11 patients were diagnosed as primary OAML but showed additional involvement of further organs. Other extranodal organs involved at diagnosis were the bone marrow in only one patient, parotid gland (n = 1), kidney (n = 2), oro-/epipharynx (n = 2), lung (n = 2), stomach (n = 2), colon (n = 1), subcutis (n = 1) and skin (n = 1). In all three patients with GI-involvement (corresponding to 5% of the total cohort) no characteristic symptoms were present and lymphoma was only seen on histology but not suspected macroscopically during endoscopy, indeed suggesting secondary involvement of the stomach. In addition during follow up, eight patients disseminated at progress/relapse including the following organs: soft tissue/subcutaneous (n = 2), lymph nodes (n = 3), lung (n = 1), kidney (n = 1), oral cavity (n = 1), epipharynx (n = 1) and stomach (n = 1). No bone marrow involvement was seen in these relapses, which - together with the single patient (1/60, 2%) having a positive marrow at diagnosis - again suggests that routine bone marrow biopsy in patients with OAML might not be necessary. None of our patients had evidence of central nervous system involvement during follow-up.

The International prognostic index (IPI) score was evaluable in 47 patients and 66% (31/47) scored as low risk, i.e. IPI 0–1. Performance status (PS) according to the European Cooperative Oncology Group (ECOG) at diagnosis was good in the majority of patients and 5% (3/60) were rated as PS>1. Beta-2-microglobulin level was evaluable in 39/60 patients and was elevated in 26% (10/39). Information on an underlying autoimmune disease was available in 47/60 patients, and 17/47 patients (36%) were positive. Chronic autoimmune thyroiditis (Hashimoto’s thyroiditis) was the most frequently diagnosed condition (7/47, 15%), two patients had Sjögren’s syndrome, one M. Bechterew/ankylosing spondylitis, one systemic lupus erythematosus and 6 tested positive for either ANA/ANCAs or rheumatic factors without clinical symptoms or a defined diagnosis. Only one of 60 patients had hepatitis B, while no evidence of infection with hepatitis C could be found in our 60 patients. Helicobacter pylori infection was present in 17 of 37 serologically tested patients (46%), which is in keeping with the rate of seropositivity in the general Austrian population. All patients with evidence of HP-infection were given antibiotic HP-eradication using a clarithromycin-based regimen plus a proton pump inhibitor.

Plasmacytic differentiation was observed in 28% (17/60), and paraproteinemia in 22% of patients (13/60). MALT specific genetic aberrations were detected in 13 of 27 patients studied (48%) and consisted of t(14;18) (q32;q21)/IGH-MALT (n = 4) accompanied by trisomy 3 in two patients, t(3;14) (p14;q32) alone (n = 1) or combined with trisomy 18 (n = 1), trisomy 3 plus trisomy 8 (n = 3) and, solely trisomy 3 (n = 3) or trisomy 18 (n = 1) respectively. For characteristics of patients see Table 1.

**Table 1:** Characteristics of patients with OAML at initial diagnosis and first line therapy.

Characteristics	No. of patients
Number of patients	60
Median FUP time in months (IQR)	43 (16–92)
Sex	
Female	33 (55%)
Male	27 (45%)
Median age (IQR)	64 (51–75)
Stage according to Ann Arbor	
IE	40 (67%)
IIE	1 (2%)
IIIE	2 (3%)
IVE (bilateral)	17 (7) (28%)
IPI score	
0–1	31/47 (66%)
>1	16/47 (34%)
Performance status	
0–1	57/60 (95%)
>1	3/60 (5%)
Clinical features	
Beta-2-microglobulin	10/39 (26%)
Paraproteinemia	13/60 (22%)
Autoimmune disorder	17/47 36%)
Hashimoto’s disease	7/47 (15%)
Pathological features	
Plasmacytic differentiation	17/60 (28%)
Genetic aberrations	13/27 (48%)
Infections	
Helicobacter pylori	17/37 (46%)
Hepatitis B	1/60 (2%)
First line therapy (n = 58)	
Local treatment (radiotherapy/surgery)	17 (14/3) (29%)
Immuno-/chemotherapy	26 (45%)
Antibiotics	9 (16%)
Wait-and-see	6 (10%)

No. = number of patients; FUP = follow up; IPI Score = international prognostic index.

### Treatment and clinical course of patients with OAML

Various forms of treatment were used in our patients and treatment data were available in 58/60 patients. Out of these 58 patients, 17 underwent local therapies, 26 systemic treatment with immuno-/chemotherapy, 9 patients were given antibiotics as first line treatment, while 6 were only watched as initial approach.

Local treatment was applied in 17 patients using either radiotherapy (24%, 14/58) or surgical resection (5%, 3/58) and resulted in response in 82% of patients (CR n = 13, PR n = 1, SD = 1, PD = 1, no data = 1). One patient progressed during radiotherapy with distant mediastinal lymph node involvement, which was diagnosed as diffuse large B-cell lymphoma by biopsy.

A total of 8 of 17 (47%) patients required further therapy; and 7 patients relapsed following CR. Two patients relapsing had recurrent orbital manifestation whereas the remaining 5 experienced distant relapse of disease. One of these patients developed contralateral OAML 21 years after CR following radiation of the right orbit, and clonal assessment of biopsies showed an identical clone. Median TTP in patients receiving local therapy was 38 months (IQR 24–53). Six out of these 8 relapses were treated systemically, two were again irradiated.

A total of 26 patients (45%) with OAML received first line systemic treatment with immuno-/chemotherapy regimens or therapy with CD20+ antibody rituximab. For detailed clinical characteristics and specifics of the various regimens used see Table 2. Fifteen patients were treated systemically in the presence of stage IE, while 11 patients had stages IIE – IVE (see Table 2). The response rate was nearly identical compared to local therapy at 85% (CR n = 16, PR n = 6, SD n = 2, PD n = 1, no data n = 1). After a mTTP of 16 months (IQR 9–31) ten patients needed further therapy. One patient was lost to follow-up before response evaluation and one patient progressed during first line systemic therapy.

**Table 2:** Characteristics of OAML patients receiving immuno-/chemotherapy as first line therapy (n = 26).

Sex	Age	Localization	Stage	PS	First linetreatment	Outcome	Progression	TTP(months)	Furthertreatment	Autoimmunedisease	FUP(months)	Alive
**M**	65	lacrimal gland LNN	IIIE	0	R-FCM	CR	no	-	no	no	44	yes
**M**	73	conjunctiva bilat.	IVE	2	R-CHOP	CR	no	-	no	ANA, RF	34	no
**M**	65	conjunctiva (parotid,kidney)	IVE	0	R-CNOP	CR	no	-	no	no	82	yes
**M**	41	conjunctiva	IE	0	R-2CdA	CR	no	-	no	Hashimoto’s disease	33	yes
**F**	66	intraorbital	IE	0	R-benadmustine	CR	no	-	no	no	32	yes
**M**	81	intraorbital	IE	1	CHOP	ND	ND	ND	ND	no	0	no
**F**	53	lacrimal gland	IE	0	R-CHOP	CR	no	-	no	systemic lupus	80	yes
**M**	54	lacrimal gland	IE	0	rituximab	SD	no	-	no	no	16	yes
**F**	74	lacrimal gland, LN	IIE	0	lenalidomide	SD	yes	28	systemic	Sjögren’s disease	29	yes
**M**	85	intraorbital (lung,stomach)	IVE	1	2CdA	CR	yes	7	systemic	no	60	no
**F**	58	lacrimal gland	IE	0	rituximab	CR	no	-	no	ANA	113	yes
**F**	51	intraorbital (colon)	IVE	1	rituximab	PR	yes	40	systemic	ANA	123	yes
**M**	53	conjunctiva bilat.	IVE	0	thalidomide	CR	no	-	no	no	146	yes
**F**	64	conjunctiva	IE	1	bortezomib	PR	no	-	no	no	82	yes
**M**	59	conjunctiva	IE	0	R-benadmustine	CR	no	-	no	no	20	yes
**M**	64	conjunctiva	IE	0	oxaliplatin	PR	yes	9	antibiotic	no	104	yes
**M**	75	conjunctiva	IE	0	lenalidomide	CR	no	-	no	Hashimoto’s disease	42	yes
**F**	79	conjunctiva	IE	0	lenalidomide	PR	no	-	no	no	17	yes
**M**	36	lacrimal gland	IE	0	bortezomib	CR	yes	9	Zevalin	Hashimoto’s disease	78	yes
**M**	87	intraorbital, LNN	IIIE	2	MCP	PD	yes	4	systemic	no	28	no
**F**	56	lacrimal gland	IE	0	bortezomib	CR	yes	44	systemic	no	73	yes
**F**	67	lacrimal gland	IE	0	lenalidomide	CR	yes	20	systemic	no	27	yes
**M**	78	intraorbital	IE	0	lenalidomide	PR	no	-	no	no	26	yes
**F**	48	conjunctiva bilat.	IVE	0	oxaliplatin	CR	yes	16	systemic	ANA	71	yes
**F**	42	intraorbital (lung,stomach)	IVE	0	bortezomib, fludarabine*	CR	no	-	no	no	74	yes
**M**	37	lacrimal gland bilat.	IVE	0	oxaliplatin	PR	yes	16	systemic	no	121	yes

*patient received concomitant radiotherapy of the orbit.

Abbreviations of therapy regimens applied: R-FCM = rituximab, fludarabine, cyclophosphamide, mitoxantrone; R-CHOP = rituximab, cyclophosphamide, doxorubicin, vincristine, prednisone; R-CNOP = rituximab, cyclophosphamide, mitoxantrone, vincristine; R-2CdA = rituximab, cladribine; MCP = melphalan, chlorambucil, prednisone, Zevalin = yttrium-90-ibritumomab tiuxetan.

PS = performance status; bilat. = bilateral, TTP = time to progression; FUP = follow up time; LN(N) = lymph node(s); CR = complete remission; PR = partial remission; SD = stable disease; PD = progressive disease; ND = no data; ANA = antinuclear antibodies, RF = rheumatic factors.

Nine patients received empirical antibiotics as initial therapy. Substances used were either doxycycline (n = 6) or clarithromycin (n = 3) and all patients except one with bilateral involvement had stage IE disease. The response rate was 38% (PR n = 1, SD n = 3, PD n = 2, no data n = 1). Remarkably, two patients achieved CR now ongoing for 6 and 82 months. Five patients needed further therapy (radiotherapy n = 2, systemic therapy n = 2, different antibiotic therapy n = 1) after 1, 2, 4, 15 and 18 months respectively.

A wait-and-see strategy was the initial approach in 6 of 58 patients and four needed consecutive systemic treatment after 17, 31, 33 and 86 months respectively.

**Figure 1:**
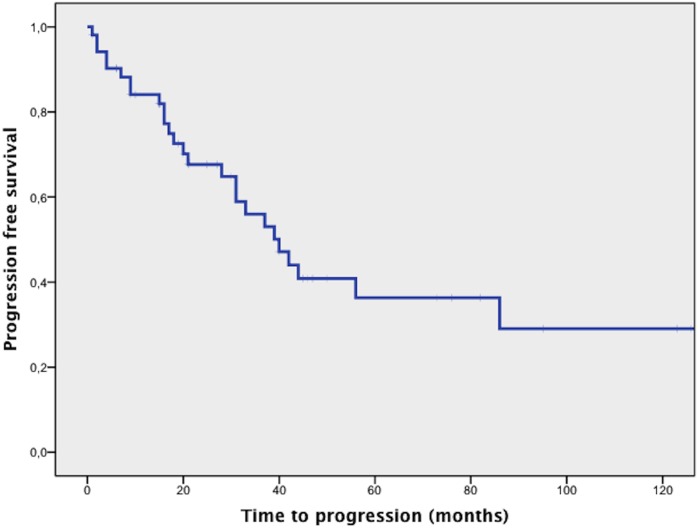
Kaplan Meier curve for progression free survival of patients with OAML treated at our institution. X-axis follow-up in months, y-axis cumulative progression-free survival.

In total 28/58 patients (48%) progressed/relapsed after first line management and were given at least one further line of therapy. Median TTP in this cohort of patients was 20 months (IQR 9–39, see Figure 1). No patient died during first line therapy. Kaplan Meier-curves for patients receiving different treatment subgroups were estimated but no significant difference in TTP after first line therapy was found (p = 0.14). There was no significant difference (p = 1.66) in the mTTP of patients with progressive disease among the different treatment subgroups when analyzed with ANOVA and no significant difference (p = 0.21) in the median follow-up time between local treatment (48 months), immuno-/chemotherapy (52 months), antibiotics (20 months) and wait and see (83 months). Furthermore, elevated beta-2-microglobulin level (p = 0.47), plasmacytic differentiation (p = 0.92), paraproteinemia (p = 0.23), presence of an autoimmune disorder (p = 0.68) or IPI score>1 (p = 0.26) were not associated with a higher risk for relapse or progress after first line therapy. Also, Kaplan-Meier curves estimated for TTP in accordance to primary site of lymphoma (i.e. conjunctiva, lacrimal gland, intraorbital localization) did not differ significantly (p = 0.12) among subgroups.

During the entire follow-up period, two patients died due to MALT lymphoma progression without evidence of transformation as assessed by repeat biopsy. Additionally in one patient, a second B-cell lymphoma (small lymphocytic lymphoma - SLL, stage IV) was diagnosed consecutively. The small lymphocytic lymphoma was clonally unrelated to the initial MALT lymphoma and treatment with rituximab and bendamustine was initiated. The lymphoma consecutively transformed to DLBCL eventually resulting in the patient’s death. In another patient infiltration with a DLBCL was found in a cervical lymph node one year after diagnosis of orbital MALT lymphoma and the patient died due to progression of this lymphoma. For characteristics of patients suffering lymphoma-related death see Table 3. Five patients died from causes unrelated to MALT lymphoma or therapy, while the remaining 51/60 patients (85%) are alive at the time of analysis. Overall survival did not significantly differ between the treatment groups (p = 0.68).

**Table 3:** Characteristics of OAML patients dying of lymphoma progression (n = 4).

Patient characteristics at initialdiagnosis	First line therapy(outcome)	Further therapiesfor OAML	Cause of death	Time from initialdiagnosis to death
male, IIIE, IPI 3, age 87	MCP (PD)	R-CHOP, radiation x2, R-DHAOx	lymphoma progression	28 months
male, IVE, IPI III, age 73	radiation (CR)	relapse at 23 months → radiation	lymphoma progression	28 months
female, IIE, IPI I, age 72	radiation (PD)	everolimus, radiation	transformation (DLBCL)	15 months
female IVE, IPI 1, age 58	doxycycline (PD)	radiation	transformation (SLL, DLBCL)	22 months

Abbreviations of therapy regimens applied: MCP = melphalan, chlorambucil, prednisone; R-CHOP = rituximab, cyclophosphamide, doxorubicin, vincristine, prednisone; R-DHAOx = rituximab, dexamethasone, high-dose cytarabine, oxaliplatin.

IPI = international prognostic index; PD = progressive disease; CR = complete remission; DLCBL = diffuse large B-cell lymphoma; SLL = small lymphocytic lymphoma.

## Discussion

Along with MALT lymphoma of salivary glands (14%), skin (11%) and pulmonary manifestations (14%), OAML accounts for one of the more frequent non-gastric localizations of this disease (12%) [4]. To the best of our knowledge this is one of the largest series of OAML to date and the follow-up of 43 months appears to be sufficiently long to assess the outcome of various first-line therapies.

Compared to the patient characteristics summed up in a recent review by Stefanovic and Lossos [12], more patients in our cohort had stage IVE disease not only with bilateral involvement but disseminated disease involving further extranodal organs. While one cannot rule out a slightly different biology or a referral bias, this might also be associated with the extensive staging-procedure implemented and used at our clinic since 1997 [28]. Thus, extensive pretherapeutic staging appears reasonable in order to choose the optimal form of management in patients with OAML. Of note is the fact that only one patient had bone marrow involvement at diagnosis, again suggesting that patients with OAML might be spared a routine bone marrow biopsy. In addition, none of our patients had central nervous system involvement during follow-up and the rate of (secondary) GI-involvement was 5% (n = 3) and in one additional patient, stomach involvement was detected in the course of disease, suggesting that routine GI-work up might not be necessary in patients with OAML.

It has repeatedly been shown that autoimmune disorders are a common feature of both extragastric as well as gastric MALT lymphoma [6]. In the present series, 36% of patients tested suffered from an autoimmune disorder, but this was not associated with a worse outcome or clinical course. Chronic autoimmune thyroiditis (Hashimoto’s thyroiditis) was the most frequent diagnosis documented. An association between thyroid orbitopathy and OAML development respectively was reported in a British series 2006 [30] suspecting this condition as a predisposing factor. In the present series, however, no patient had auto-antibodies or symptoms indicating Grave’s disease. Other previously discussed risk factors evaluated, e.g. elevated beta-2-microglobulin, plasmacytic differentiation, exact localisation of OAML (i.e. lacrimal gland, conjunctival or intraorbital) and presence of paraproteinemia also did not affect the prognosis in our cohort of patients.

Ocular MALT lymphoma has repeatedly been correlated with an excellent prognosis even compared with other indolent lymphomas of the ocular adnexa e.g. follicular lymphoma [31]–[33]. In view of the indolent behaviour of the disease along with a usually non- or oligo-symptomatic course, not only the efficacy but also possible side effects should be especially considered in the therapeutic decision. Local therapy with either radiation or surgery (e.g. in conjunctival lymphoma) has shown excellent local control with low relapse rates although re-occurrence of the disease seemed to be more frequent in initially operated patients [12]. Thus, local therapy as first-line treatment for limited stages OAML has given excellent results in various studies, although it has not yet been directly compared to systemic treatment approaches. Radiotherapy with an investigator-depending dose of 20–45 Gy achieved high response rates with complete remissions in up to 100% of patients in most studies [11]–[13], [34], [35]. Son et al [36] have reported on a series of 46 patients treated with radiotherapy and have defined a dose of 30.6 Gy applied in fractions of 1.8–2.0 Gy to be a reasonable cut-off in terms of tolerability and efficacy. In a Japanese study of 78 patients, a total of 23 (30%) subsequently developed grade III cataract and 7 patients had retinal disorders [37]. A recent study has compared directly the ophthalmologic outcomes of radiotherapy versus chemotherapy or combined chemo-radiotherapy and has demonstrated significantly lower rates of ophthalmological symptoms in patients with non-conjunctival OAML receiving no radiation therapy (p = 0.02, median follow up time 30 months) [11] while the therapeutic outcome was not impaired.

In has to be noted, however, that the retrospective nature of our evaluation does not allow for a disctinct assessment of criteria decisive for the use of respective therapies in individual patients. In addition, also patients treated with non-approved drugs during registered clinical trials initiated at our institution were included in our analysis, adding some potential bias to our data. With all the caveats of such a retrospective series, there appeared to be no difference between local therapies as compared to systemic approaches. First line therapy with radiotherapy, surgery or immuno-/chemotherapy resulted in comparably high response rates (82% vs 85%), which also did not result in a significantly different TTP between treatment arms. However, there was no standardized systemic regimen, which might weaken the conclusion of our series (see Table 2). In addition, also new and investigational substances/regimens as lenalidomide or bendamustine were used in patients included in clinical trials [19], [38]. However, still these data allow for the hypothesis that the wide variety of systemic approaches used in clinical everyday practice did apparently not result in a worse outcome than radiotherapy. Hashimoto and coworkers [37] presented long-term outcomes of 78 OAML patients treated with radiotherapy. Local control rates were excellent as no patient had locally recurrent disease, and 10 patients (13%) relapsed at a distant site. Interestingly, 20 of those 78 patients received not only radiotherapy but combined treatment with immuno-/chemotherapy and none of these 20 patients had systemic recurrence, suggesting a potential benefit of systemic treatment in OAML. In addition it has been suggested that MALT lymphoma is a disseminated diseases in up to one third of patients at diagnosis and relapses may occur even after a prolonged period of time [14], [39].

Several recent prospective and retrospective series have dealt with antibiotic therapy of OAML. Especially data reported by Ferreri and coworkers [23], [26], [27] have repeatedly demonstrated the efficacy of eradication therapy with doxycycline 200 mg daily resulting in high response rates up to 45–65%. In addition, Govi et al [24] presented relapsed/refractory OAML treated with clarithromycin showing equal response rates on a potential background of direct antitumor activity as supposed for macrolides. However, strong geographic variations of this bacteria have been reported in the past [9] and eradication therapy was equally effective in CP negative patients in some series [23], [25] as also highlighted in a review recently published by our institution [40]. In the present work nine patients were treated up-front with doxycycline or clarithromycin and two complete remissions were achieved though overall response rate was rather low (38%) when compared with other treatments. However, significant side effects of antibiotic therapy are extremely rare and the median follow up time in this group of 20 months is still relatively short.

Six patients refused initial therapy; two were lost-to follow up due to a lack of compliance and the remaining four needed systemic treatment after a mTTP of 32 months. This long period, as well as the absence of severe complications/symptoms occurring in the period of non-treatment, suggest that a wait-and-see policy might be a reasonable option in some patients if affection of the optic nerve can be ruled out upfront by imaging and broad ophthalmological tests. This was also emphasized by Tanimoto and colleagues [22], who have only observed 36 asymptomatic patients with localized OAML. After an impressive median follow-up of 7 years, approximately 70% did still not require treatment, while progression was noted in 47% and 6% died due to progressive lymphoma.

Taken together, the current retrospective series of 60 patients reflects the excellent prognosis, but also the heterogeneous nature of OAML under “daily-life” conditions. There was no significant difference in TTP and response rates between local vs systemic therapy, and also wait and see as well as antibiotics appeared to be sensible options in selected patients. In view of our data, we strongly suggest that a randomized trial of radiation versus systemic therapy in patients requiring therapy is warranted to answer the question of optimal management. In the clinical practice, however, our data suggest that OAML is an idolent disease irrespective of stage and does not require immediate therapy in a large percentage of patients. In the absence of randomized studies, various forms of therapy appeared to be equally effective in our analysis. This suggests that the individual management should be based on minimizing toxicities and also take in to account the respctive center’s experience with various forms of therapy along with patients’ preferences.
